# Novel endoscopic gastric purse-string suture device for weight management in a porcine model (with video)

**DOI:** 10.1055/a-2539-9167

**Published:** 2025-04-15

**Authors:** Yiyu Qiao, Shuqian Hu, Wei Shen, Airong Tang, Xueting Zhang, Hanqing Zhang, Min Min, Yan Liu

**Affiliations:** 1 26460 Department of Gastroenterology, the 5th Medical Center of PLA General Hospital, Beijing, China; 2 12538 The School of Medicine, Nankai University, Nankai District, Tianjin, China; 3 678390 Department of Gastroenterology, Senior Department of Gastroenterology, the 1st Medical Center of PLA General Hospital, Beijing, China

**Keywords:** Quality and logistical aspects, Performance and complications, Training, GI surgery

## Abstract

Obesity is a global health issue. Traditional treatment modalities such as lifestyle modifications, pharmacological interventions, and bariatric surgery often present various limitations. Recently, endoscopic bariatric and metabolic therapies (EBMTs) have emerged as promising minimally invasive approaches for weight management. These therapies reduce gastric volume and energy intake, prolong gastric emptying, and regulate hormones associated with satiety. This study evaluated endoscopic gastric purse-string suturing (EGPSS), a novel endoscopic procedure designed to reduce gastric fundus volume while minimizing associated risks. We assessed its feasibility and efficacy in a porcine model in terms of histological, physiological, and metabolic outcomes. The results suggest that EGPSS offers a viable option for metabolic control.

## Introduction

Obesity is a global epidemic with a significant impact on health, leading to an increased risk of conditions such as type 2 diabetes, cardiovascular disease, and non-alcoholic fatty liver disease. Traditional treatment options, including lifestyle modifications, pharmacological therapies, and bariatric surgery, have limitations related to their efficacy, invasiveness, and potential for adverse events (AEs). In recent years, endoscopic bariatric and metabolic therapies (EBMTs) have emerged as promising, minimally invasive alternatives that effectively manage obesity and associated metabolic disorders 1 . These therapies work by reducing gastric volume and energy intake, stimulating mechanical and chemical receptors in the stomach, and regulating hormones that modulate appetite and satiety.

**Fig. 1:**
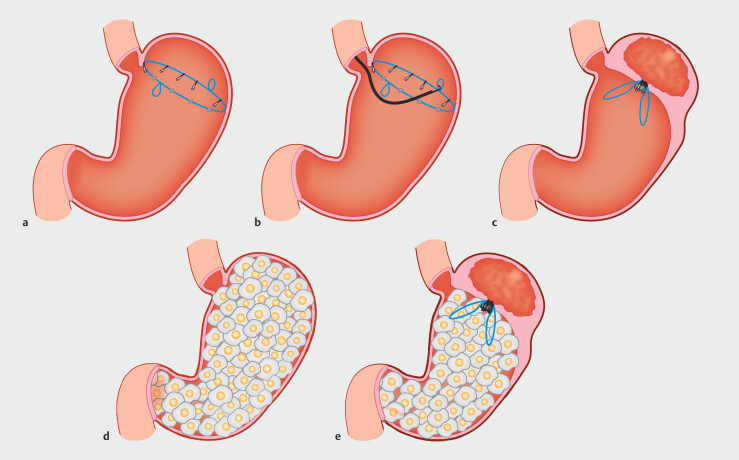
**a** Clips are used to fix the endoloop at the stomach fungus, avoiding the esophagus. **b** An endoscope hook is used to tighten the endoloop. **c** The mucous membrane is drawn inward to form a pouch. **d** Before surgery, food can fill the gastric fundus. **e** After surgery, the stomach cavity has been significantly reduced in size.

Among the different EBMTs, techniques such as intragastric balloon therapy, endoscopic sleeve gastroplasty (ESG), aspiration therapy, and duodenal-jejunal bypass liners are commonly used 2 3 4 . While some of these techniques, such as ESG, have demonstrated effective results, they often involve complications such as mucosal damage, perforation, infection, and bleeding. To address these concerns, we developed endoscopic gastric purse-string suturing (EGPSS), a novel method designed to reduce gastric fundus volume while minimizing risks. EGPSS uses a specially designed endoloop and endoclip system to aggregate the gastric mucosa ( Fig. 1 ), offering a potential short-term weight management approach with fewer complications. The purpose of this study was to evaluate the feasibility and efficacy of EGPSS in a porcine model, focusing on histological and physiological outcomes.

## Methods

### Animal ethics statement

The experimental protocol was reviewed and approved by the Institutional Animal Care and Use Committee (IACUC) of the animal testing institution to ensure compliance with ethical standards established by the Chinese Animal Committee. All procedures adhered to the guidelines outlined in the Guide for the Care and Use of Laboratory Animals 5 and the ARRIVE guidelines (Animal Research: Reporting of In Vivo Experiments).

### Materials and design

**Fig. 2:**
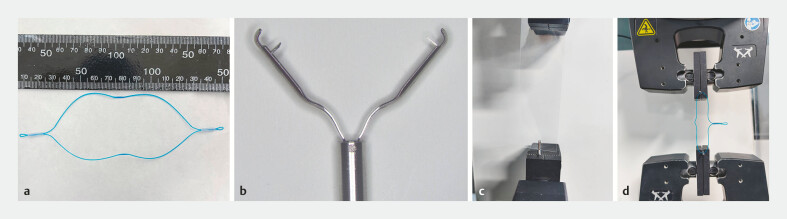
**a** Photograph of an 8-cm endoloop. **b** Photograph of a novel endoclip. **c** Photograph of the clip pulling strength assessment. **d** Photograph of the endoloop tension test.

To meet the dimensional requirements for gastric suturing, we developed a dual-tail endoloop. The endoloop is made from polydioxanone monofilament and consists of two plastic tubes on either side, which facilitate closure but prevent reopening. The loop measures approximately 10 cm by 4 cm in its open state ( Fig. 2 **a** ). When tightened, the two free tails are securely locked in place, ensuring adequate aggregation of the gastric mucosa.

To meet the tension requirements of the dual-tail endoloop, we also designed a new type of endoscopic clip. The clips are made of metal, with a double-pronged structure similar to traditional clips. However, the jaw structure has been modified with two teeth measuring 1.4 mm in length to enhance gripping strength. In addition, the clip head is polished to minimize pressure on the clamped tissue, and the gap between the arms allows for sufficient blood flow, thus reducing tissue ischemia and prolonging clip retention ( Fig. 2 **b** ).

#### Endoscopic clip pulling strength test

We tested the clips on a 0.35-mm-thick polyurethane film (100 mm long, 30 mm wide) according to the Chinese standard (YY/T 0282–2009). One end of the film was fixed to a base and the metal tail of the clip was placed on the micromechanical testing system. We measured the pulling or holding force until the clip slipped. Ten new endoclips and 10 traditional clips (L2716195, LeoMed, Changzhou, China) were tested ( Fig. 2 **c** ).

#### Endoloop tension test

When pulling the dual-tail endoloop, it loosens once the pulling force exceeds the tension between the loop and the plastic tube. The loop fully loosens when the force consistently exceeds tube tension. To measure maximum tension, we used eight 4-cm single-tail endoloops to avoid interaction between the two tails. Both ends of the endoloops were secured to the testing system and we gradually increased the force, recording the force when the first slippage or breakage occurred ( Fig. 2 **d** ).

### Surgical procedure

Step-by-step demonstration of endoscopic gastric volume reduction using multiple novel endoscopic clips and endoloop. This technique enables effective tissue approximation and intraluminal plication for minimally invasive gastric remodeling.Video 1

Eight 5-month-old female Bama miniature pigs were selected for the study. The pigs were fasted for 48 hours before the procedure and their preoperative body weights were recorded. The animals underwent general anesthesia and mechanical ventilation. A dose of 5 mg/kg of Schutai 50 (tiletamine hydrochloride and zolazepam hydrochloride) was administered intramuscularly for anesthesia induction. After tracheal intubation, anesthesia was maintained with 2% isoflurane. Blood samples were collected to assess total cholesterol, glycated hemoglobin (HbA1c), and other nutritional indicators. All procedures were performed by two experienced endoscopists using a standard endoscope (EG-580RD, Fuji).

The pigs were divided into two groups: one group (n = 2) underwent a mucosal incision, and the other group (n = 6) received a non-incision procedure. In the incision group, the gastric mucosa was incised to expose the muscle layer with a dual knife (KD-650L, Olympus Corporation, Tokyo, Japan). A dual-tail endoloop was introduced via endoscopic forceps and secured with 14 endoscopic clips. In the non-incision group, clips were placed directly on the gastric mucosa without need for an incision (Supplementary Fig. 1). The endoloop was tightened with an endoscope hook to aggregate the mucosa, and its effectiveness was assessed through endoscopic follow-up ( Video 1 ).

### Efficacy assessment

**Fig. 3:**
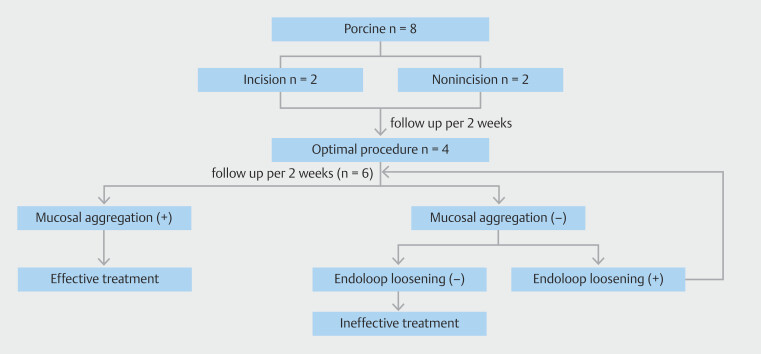
Flowchart of the experiment.

**Fig. 4:**
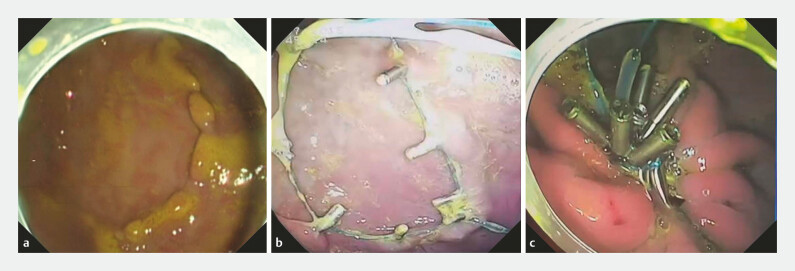
**a** All the clips and endoloops detached, and mucosal aggregation was completely lost. **b** The endoloop was partially loosened, and mucosal aggregation was lost. **c** The endoloop remained tight, and mucosal aggregation persisted.

Therapeutic efficacy was evaluated based on sustainability of the procedure during follow-up ( Fig. 3 and Fig. 4 ). We monitored the pigs for up to 8 weeks and recorded weight changes, as well as metabolic markers (HbA1c, total cholesterol). Histological evaluation of the gastric mucosa was performed at the end of the study. Euthanasia was performed and tissue samples were collected for further examination.

### Statistical analysis

Data were analyzed using SPSS 21. Results were expressed as means ± standard deviations or medians (ranges). Independent sample *t* -tests were used to compare differences between groups and a *P* value < 0.05 was considered statistically significant.

## Results

### Mechanical performance

In the mechanical performance testing, the endoscopic clips showed a maximum pulling force of 2.46 ± 0.13 N, significantly stronger than the traditional clips, which exhibited a maximum pulling force of 0.27 ± 0.09 N. The dual-tail endoloop also demonstrated a high tensile strength, with an average initial sliding force of 2.02 ± 0.09 N and an average pulling force during breakage of 31.82 ± 1.47 N (Supplementary Fig. 2).

### Follow-up outcomes

#### Procedure specifics

In the incision group, average procedure duration was 55 minutes, whereas the non-incision group took 20 minutes. Minor bleeding was observed in two pigs in the incision group, which was controlled effectively. However, by 4-week follow-up, both pigs in the incision group experienced treatment failure due to detachment of clips and endoloops. In contrast, both pigs in the non-incision group maintained stable outcomes ( Table 1 ).

**Table:** **Table 1** Treatment effectiveness.

	Ineffective treatment	Effective treatment	Supplementary treatment
	Mucosal aggregation (–) endoloop loosening (–)	Mucosal aggregation (+) endoloop loosening (–)	Mucosal aggregation (–) endoloop loosening (+)
P1*	4 weeks		
P2*	4 weeks		
P1	8 weeks		
P2	6 weeks		
P3		8 weeks (12 weeks*)	4 weeks
P4		8 weeks (12 weeks*)	4 weeks
P5		8 weeks (12 weeks*)	4 weeks
P6		8 weeks	
*12-week total follow-up time of primary and supplementary treatment.			

#### Surgical effectiveness

In the non-incision group, the pigs were monitored for up to 8 weeks. Two pigs required supplementary treatment after 6 weeks due to partial loosening of the endoloops (Supplementary Fig. 3). After the supplementary procedure, the gastric mucosa remained aggregated. Histological analysis revealed no significant damage to the gastric mucosa and the clips were successfully removed at the end of the study.

### Metabolic outcomes

**Fig. 5:**
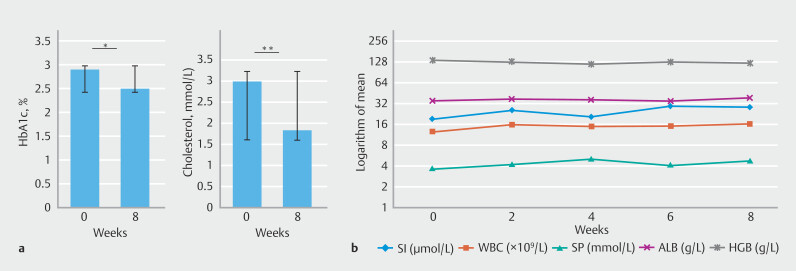
**a** Significant reductions in average hemoglobin A1c (HbA1c) ( *P* = 0.007) and total cholesterol ( *P* = 0.000). **b** No observed complications such as electrolyte imbalances, hypoalbuminemia, or anemia.

At the 8-week follow-up, there was a significant reduction in levels of both HbA1c ( *P* = 0.007) and total cholesterol ( *P* = 0.000) ( Fig. 5 **a** ), indicating improved metabolic health. No significant overall average weight loss trend was observed (Supplementary Fig. 4). No complications such as electrolyte imbalance, hypoalbuminemia, or anemia were observed in the study ( Fig. 5 **b** ).

### Histological findings

Histological examination of the gastric tissue at the suture site showed intact mucosa, with no signs of inflammation, fibrosis, or necrosis. Peritoneal vessels remained intact, and there was no evidence of tissue damage or hemorrhage (Supplementary Fig. 5).

## Discussion

EGPSS is a promising alternative to traditional bariatric surgeries. The technique is performed through natural orifices, minimizing risks associated with invasive surgery. Compared with laparoscopic sleeve gastrectomy, EGPSS is quicker, less invasive, and associated with fewer complications, such as infection and bleeding. Reversibility of the procedure also allows for adjustments if needed.

Our study demonstrated the effectiveness of the dual-tail endoloop in securing the gastric mucosa. Use of a dual-tail endoloop for mucosal suturing not only reduces the number of endoscopic clips but also results in a larger suture area than does use of a single-tail endoloop (Supplementary Fig. 6). The modified endoscopic clips provided strong retention, with most clips remaining in place for more than 6 weeks. In addition, we considered the following options to address the issue of loosening of the endoloops during follow-up. First, after the endoloop has been tightened, we used another endoscopic clip to secure the root of the endoloop. Second, we modified the endoloop, such as increasing the length of the plastic tube to increase friction or using a barbed loop to prevent loosening. Finally, considering the existence of the feeding reflex in humans, maximum tension that can be formed in the stomach is less than that in the pig model, so the endoloop is less likely to loosen in a human model. This finding will be further verified in a human model.

The key advantage of EGPSS over other EBMTs is its minimal invasiveness. Techniques such as intragastric balloon and ESG are associated with AEs such as pyloric obstruction and gastric wall damage 6 7 8 9 10 , whereas EGPSS focuses only on the mucosal layer, avoiding these complications. The procedure offers a short-term weight management solution with fewer risks and complications.

To some extent, this study revealed that this endoscopic procedure has a certain effect on blood glucose and total cholesterol in porcine models. Although weight loss was not significant, reductions in HbA1c and cholesterol levels suggest that EGPSS can improve metabolic health, particularly in patients with obesity-related conditions However, owing to differences between pigs and humans, there are certain challenges in application of this surgery to humans. First, the gastric wall is significantly thicker in pigs than in humans, which leads to a prolonged retention time of the clips in humans. The duration of the surgical effect needs to be reevaluated in humans. In addition, the human feeding reflex may contribute to the ability of obese patients to lose weight in the short term. Large-scale human trials are needed to confirm the impact of this treatment on weight loss.

## Conclusions

EGPSS is an effective, safe, and reversible technique for short-term weight management and metabolic control in animal models. It shows promise as a minimally invasive option for patients with mild obesity or those requiring temporary metabolic improvement. Further studies, including large-scale human trials, are necessary to confirm its efficacy and safety in clinical settings.

## Supplementary Material

Supplementary Material Zusatzmaterial
