# Supplementary material for: Novel endoscopic gastric purse-string suture device for weight management in a porcine model (with video)
Source: Endosc Int Open. 2025 Apr 15;13:a25399167. doi: 10.1055/a-2539-9167 (PMC12042993; doi:10.1055/a-2539-9167)

Supplementary Fig. 1 a When a submucosal incision is performed to expose the muscular layer, an endoscope clamp is used to fix an endoloop onto the muscular layer, and tightening is finished via an endoscope hook**. b** Without a mucosal incision, the endoloop is fixed directly to the gastric mucosa via endoscope clips.

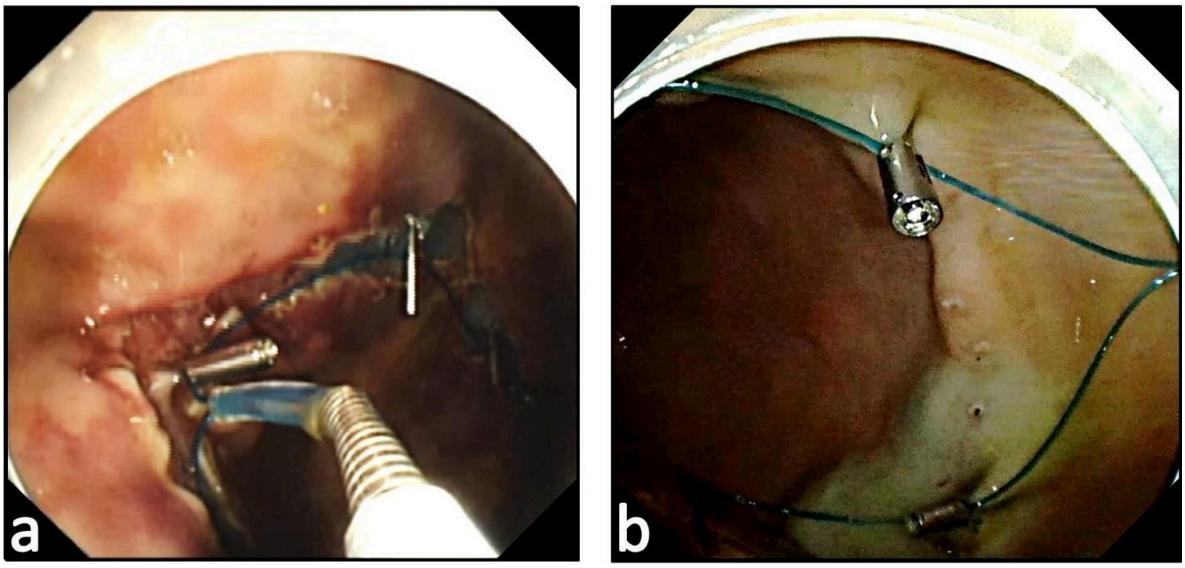

Supplementary Fig. 2 a The mechanical test data for the pulling forces of novel and traditional clips. **b** The mechanical test data for the pulling force of the 4-cm endoloop.

a

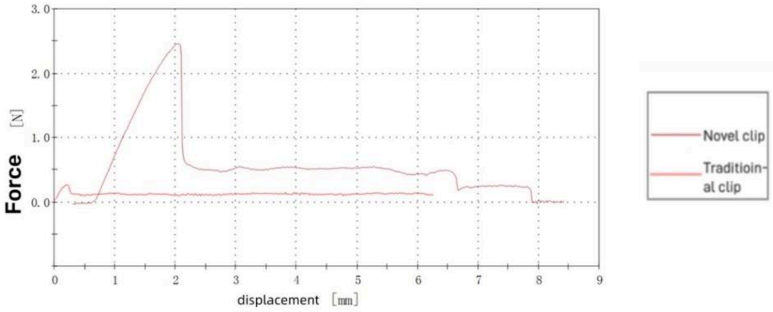

	Maximum Pulling Force(N)	Displacement at Max Force(mm)
Traditional clip	0.27	0.21693
Novel clip	2.46	1.73347

b

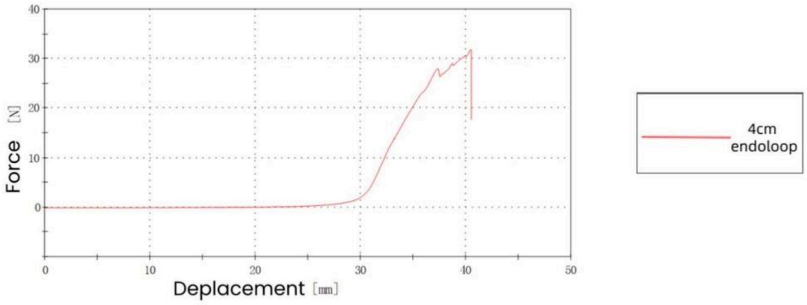

	Maximum Pulling Force(N)	Displacement at Max Force(mm)
4cm endoloop	31.82	40.43386

Supplementary Fig. 3 Each box in the table shows the remaining (Y) or dislodging (N) of the endoloop in four quadrants of the surgical area.

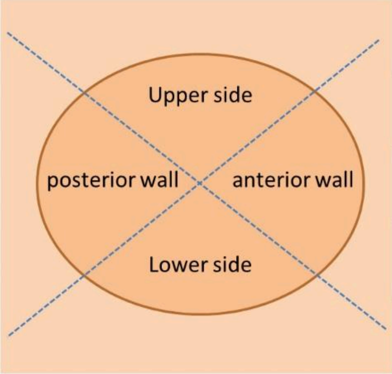

Four Quadrants of the Surgical Field – Model	P1*	P2*	P1	P2	P3	P4	P5	P6
0-week	Y, Y, Y, Y							
4-week	N, N, N, N	N, N, Y, Y	Y, Y, Y, Y	Y, Y, Y, Y	N, Y, Y, Y	Y, N, Y, Y	Y, N, N, Y	Y, Y, Y, Y
6-week	/	/	Y, Y, Y, Y	N, N, Y, N	Y, Y, Y, Y	Y, Y, Y, Y	Y, Y, Y, Y	Y, Y, Y, Y
8-week	/	/	N, N, Y, Y	/	Y, Y, Y, Y	Y, Y, Y, Y	Y, Y, Y, Y	Y, Y, Y, Y
12-week	/	/	/	/	Y, Y, Y, Y	Y, Y, Y, Y	Y, Y, Y, Y	/

Supplementary Fig. 4 Changes in the weights of the eight pigs.

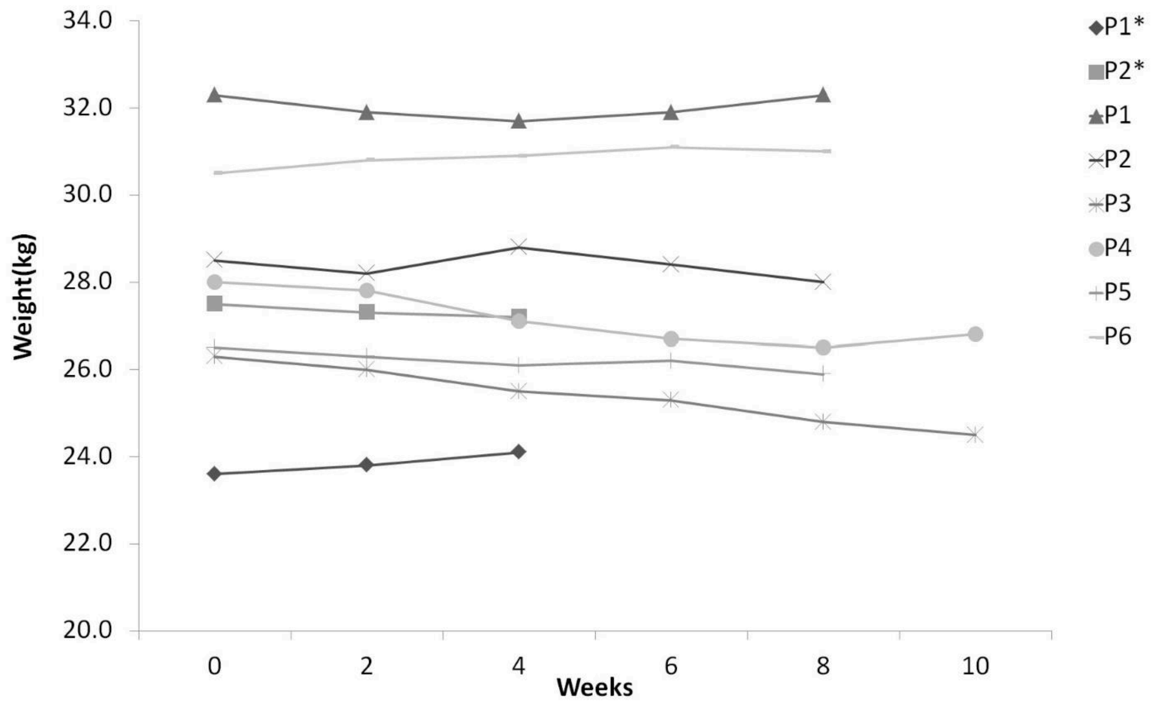

Supplementary Fig. 5 a No obvious evidence of mucosal or submucosal inflammation or fibrosis was observed within the suture area. **b** the submucosal layer and mucosa outside the suture area showed no apparent signs of inflammation or fibrosis, with the occasional presence of lymphoid follicles.

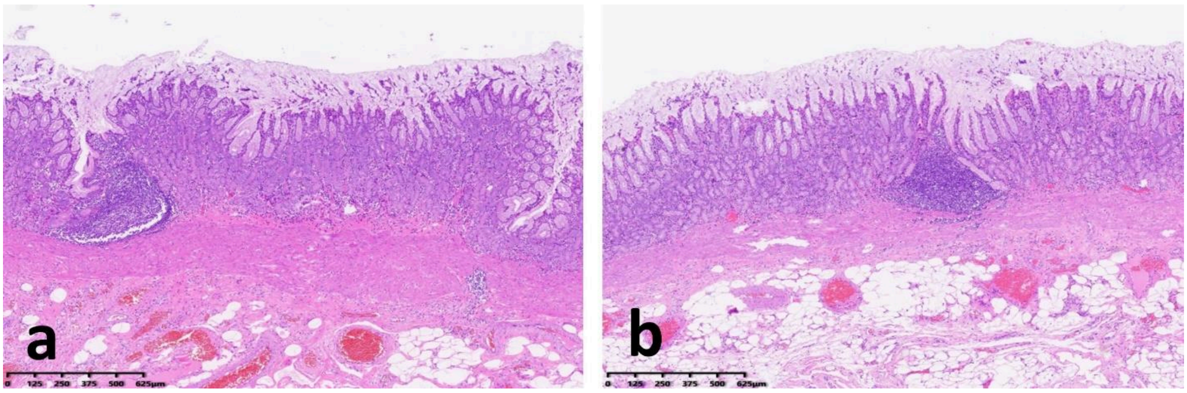

Supplementary Fig. 6 The square of a dual-tail endoloop is approximately 10 x 4 cm^2, approximately 1.5 times greater than that of a single-tail endoloop.

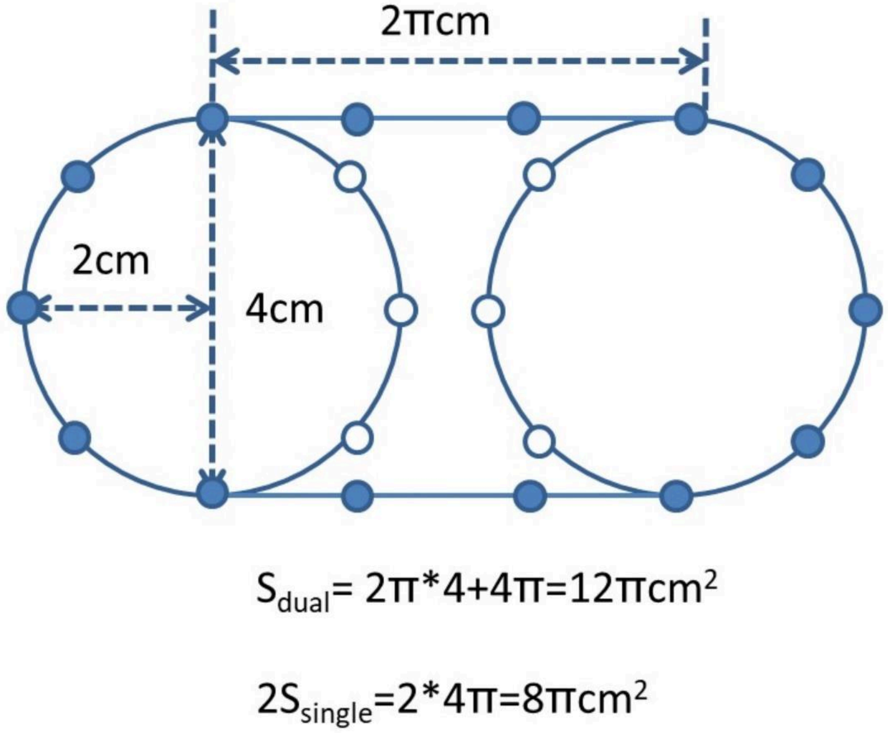

Supplement: Supplementary file 2 — Supplementary Material [file 10-1055-a-2539-9167_25428609.pdf]
